# Static and Dynamic Magnetization of Gradient FeNi Alloy Nanowire

**DOI:** 10.1038/srep20427

**Published:** 2016-02-11

**Authors:** Haozhe Yang, Yi Li, Min Zeng, Wei Cao, William E. Bailey, Ronghai Yu

**Affiliations:** 1 School of Materials Science and Engineering, Beihang University, Beijing 100191, China; 2 Materials Science Engineering, Department of Applied Physics Applied Mathematics, Columbia University, New York 10027, USA

## Abstract

FeNi binary nanowires with gradient composition are fabricated by the electrodeposition method. The energy dispersive spec-trometer line-sweep results show that the composition changes gradually along the wire axis. The gradient FeNi nanowires exhibit polycrystalline and crystal twinning at different areas along the nanowire axis, with a textured face-centered cubic structure. The static and dynamic magnetization properties are characterized by a hysteresis loop and ferromagnetic reso-nance with pumping frequencies from 12– 40 GHz. The linear dispersion of the pumping frequency *vs*: the resonance field has been observed with the applied bias field higher than the saturation field, corresponding to the hysteresis loop. The field-sweep linewidths decrease with increasing pumping frequency, and the frequency-sweep linewidths stay nearly constant at the unsaturated region. The linewidth is a Gilbert type at the saturated state, with damping of 0.035 ± 0.003. Compared with the damping of the homogeneous composition FeNi nanowire (a = 0.044 ± 0.005), the gradient FeNi nanowire may have less eddy current damping, which could make it an alternative candidate for spintronics and microstrip antennas.

One-dimensional metallic and alloy nanostructures have been extensively studied in recent years because of their fantastic properties of the surface effect, small size effect, quantum effect and macro-quantum tunnel effect^1^. Ferromagnetic nanowires are of special interest because of their fundamental importance and potential applications in high-density magnetic memories, high-frequency applications and domain wall-based logic and memory devices^2^,^3^. Compared with traditional high-frequency ferrites, ferromagnetic nanowires have higher saturation magnetization and shape anisotropy, which can provide higher permeability according to Snoek’s law^4^. The ferromagnetic nanowire array, embedded into a porous template, is a promising candidate material for self-biased microwave and millimeter-wave applications^1^ because the ferromagnetic resonance (FMR) is observed in the microwave frequency range under zero external magnetic fields. The magnetic anisotropy of a ferromagnetic nanowire can be adjusted by controlling the crystal structure^5^, composition^6^ and intrinsic exchanging coupling^7^. By applying an external magnetic field, these magnetic properties can be easily tuned over a wide range of frequencies for a wider application.

Previous papers have reported that the wire density, the phase of the material and the composition of nanowires can tune the resonance frequency over large ranges^6^,^8^,^9^. However, more complex microwave responses for magnetic nanowire arrays are needed in order to expand the material and device functionality.

Gradient composition materials, such as films or bulk, play important roles in the fields of many functional materials like shape memory alloys or semiconductors^10^,^11^,^12^. Because of the wide gradation of physical and chemical properties, the crystal structure and orientation are regulated, which then affects the magnetic properties of nanowires^13^.

Therefore, we attempted to fabricate a binary alloy nanowire consisting of gradient binary compositions by electrode deposition to study the gradient composition dependent phenomena, which may inspire the research and application for this new structure. FeNi alloy shows a variety of physical characteristics when the chemical ratio is changed, and it is one of the universal soft magnetic materials with extensive application. The magnetic properties of homogeneous FeNi nanowires have been studied extensively^14^,^15^. However, the FeNi gradient composition nanowire has not yet been studied. As an effective method for obtaining highly ordered magnetic nanostructures, porous anodic aluminum oxide (AAO) assisted electrodeposition can change the morphology and compositions of the nanowires. In this paper, we describe the fabrication FeNi alloy nanowires with a gradient chemical ratio in the AAO template by electrodeposition. The morphology, composition, and magnetic properties of gradational FeNi alloy nanowires are investigated. Here, we used the ferromagnetic resonance technique (FMR) along with the hysteresis loops measured with a vibrating sample magnetometer (VSM) to give the average magnetic anisotropy of the nanowire arrays, as it can provide information on the magnetization, magnetic anisotropy and damping in magnetization dynamics^16^,^17^,^18^,^19^.

## Results

### Structure Analysis

**Figure 1:**
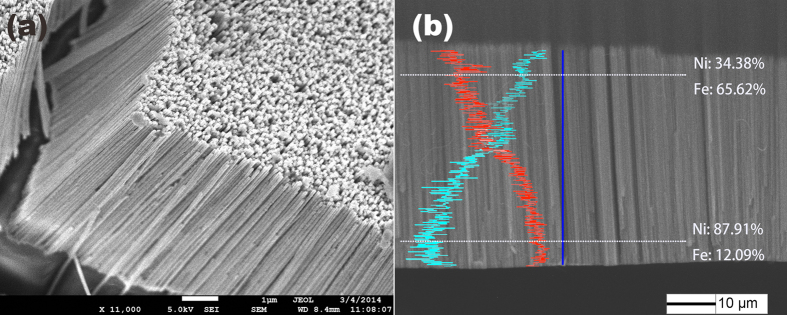
(**a**) Top-view SEM image of the gradient FeNi gradient nanowire. (**b**) Cross-sectional view SEM image and SEM elemental line scan image of Co and Ni (the blue spectra represent Ni, and the red spectra are Fe).

A scanning electron microscope (SEM) is used to observe the elemental distribution along the longitudinal direction. A top view of the gradient FeNi gradient nanowire array is shown in Fig. 1(a). The nanowires are continuous and uniform throughout the entire length, highly homogeneous in shape and arranged roughly parallel to each other. The length of the nanowire is approximately 40 *μm*. Figure 1(b) includes the cross-sectional SEM micrograph and the corresponding elemental line-scanning image. The elemental line-scanning indicates the gradient distribution of Ni and Fe. At the bottom of the nanowire, the amount of Ni atoms (87.91%) is larger than the amount of Fe atoms. The elementary ratio of Ni decreases to 34.38% at the top of the nanowire. The Fe atom has the opposite distribution tendency compared with Ni, exhibiting atom ratios of 12.09–65.62% from the bottom to the top.

**Figure 2:**
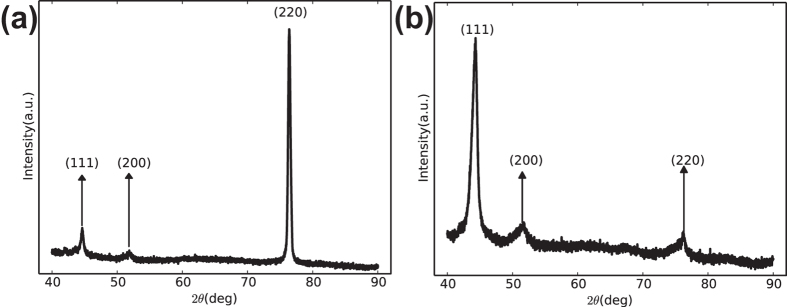
XRD spectra of (**a**)the gradient FeNi nanowire and (**b**) the homogeneous FeNi nanowire.

To compare the crystal structure between the gradient and homogeneous nanowire, we measured X-ray diffraction (XRD). Figure 2(a,b) display the XRD diffraction pattern of the gradient and homogeneous FeNi nanowire, respectively, without removing the AAO template. Considering the three peaks at 2*θ* = 44.5° (111), 51.8°(200) and 76.4°(220), the crystal structure of both kinds of FeNi nanowires can be confirmed as face-centered cubic (fcc). The relative intensity of the diffraction peaks for the gradient nanowires shows (110) the preferred orientation. This textured structure has also been reported by other researchers in Ni and FeNi nanowire arrays with high extra-uniaxial anisotropy^20^,^21^. The highly textured structure may change the magneto-crystalline anisotropy of the nanowire, which is discussed below.

**Figure 3:**
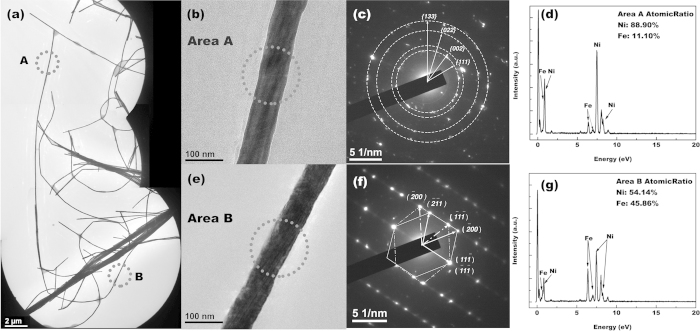
(**a**) Low-magnification TEM image of the gradient FeNi nanowires; (**b**,**c**) high-magnification TEM image of the Ni-rich area and the corresponding SAED pattern; (**d**) the EDS spectra of the corresponding area A; (**e**,**f**) high-magnification TEM image of the Fe-rich area and the corresponding SAED pattern; and (**d**,**g**) the EDS spectra of the corresponding areas A and B.

The EDS analysis, along with transmission electron microscopy (TEM), can confirm the chemical ratio of the micro area composition for a single gradient nanowire. The AAO templates were dissolved into KOH aqueous solution before TEM observation, as in our previous reports^22^. The TEM image in Fig. 3(a) is a combination of three individual images with the same magnification. An unbroken FeNi gradient nanowire is selected, with two micro areas (Fig. 3(b,e)) selected to analyze the chemical ratio. As shown in Fig. 3(a), the single bending FeNi nanowire has a length of approximately 37 *μ*m, corresponding to the cross-sectional SEM image shown in Fig. 1(b). The high-magnification TEM images for the two areas are shown in Fig. 3(b,e), exhibiting a uniform diameter of approximately 80 nm. According to the corresponding EDS results shown in Fig. 3(d,f), area A has a Ni atom ratio of 88.9% and an Fe atom ratio of 11.1%. Area B has 65.02% Ni and 34.98% Co and is 28 *μ*m away from area A.

Selected area electron diffraction (SAED) patterns for micro areas A and C are shown in Fig. 3(c,f), respectively. The Ni-rich area, corresponding to Area A, shows a polycrystalline fcc SAED pattern. The diffraction rings correspond to the (111), (002), (022) and (133) crystal planes. The polycrystalline phenomenon was also observed in homogeneous FeNi nanowires by Liu^6^. However, the low-Ni area (Area B) exhibits two single-crystal fcc SAED patterns. Furthermore, the two sets of single crystal structures share a same (111) crystal plane, showing a twinning crystal structure. The SAED result proves the formation of the *γ−* FeNi (fcc) structure and is consistent with the XRD results. Additionally, the SAED pattern indicates the different crystallinity of the FeNi nanowire with changing ion concentration in the electrolyte. The nanowire tends to form smaller grains in the Ni-rich electrolyte, corresponding to the polycrystal area. When improving the Fe^2+^ concentration in the electrolyte, the nanowire tends to form a twinning crystal structure, which has a larger grain size than the Ni-rich area. It has been reported^6^ that the electro-chemical deposition Fe_1−*x*_Ni_*x*_ nanowire tends to have a mixed phase of bcc and fcc in the range of 0.35 < x < 0.5 and only an fcc phase for x > 0.5. This is because the deposit rate of Fe^2+^ is higher than Ni^2+^, and the bcc structure (Fe) is more easily formed. In the gradient FeNi nanowire, the two mixed phase coexistence is not observed compared to the homogeneous nanowires, which can probably be attributed to the following reasons. First, the electrolyte has only Ni^2+^ when the deposition starts, which makes the nanowires form the fcc (Ni) structure. The nanowires may tend to form the origin structure (fcc) as the deposition continues and then form an fcc solid solution by adding the Fe^2+^ to the electrolyte. Secondly, the electrolyte may have a higher concentration gradient in the pores of the AAO template compared with that when depositing the homogeneous FeNi nanowires. This may give a smaller bcc crystallization driving force because of the high concentration gradient.

### Magnetic Properties

**Figure 4:**
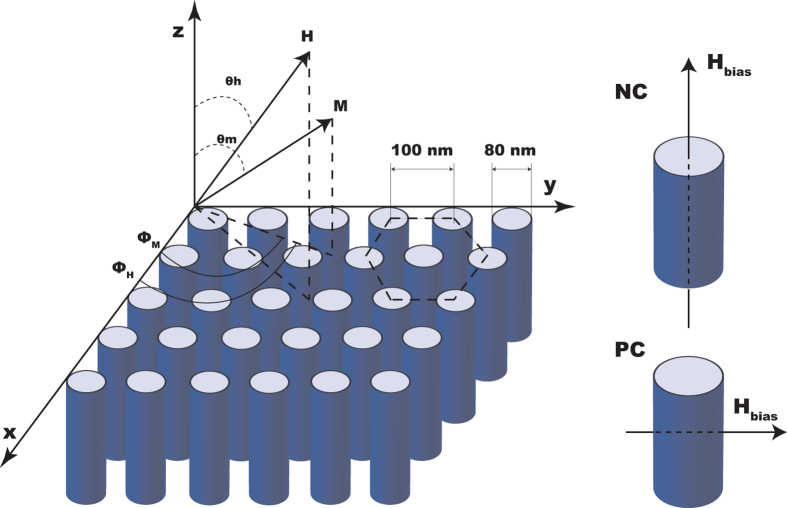
Schematic of the arrays of the magnetic nanowires, the relative orientations of the equilibrium magnetization M and the bias magnetic field H.

The sample geometry, relative of the equilibrium magnetization M, the applied bias field H, and the experimental coordinate systems of both FMR and VSM are shown in Fig. 4. In this paper, NC (bias field normal to the plane condition) stands for the parallel-nanowire-axis condition and PC (bias field parallel to the plane condition) stands for the perpendicular-nanowire-axis condition.

The free-energy density equation^23^ for a homogeneously magnetized magnetic nanowire can be written as


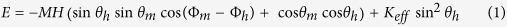


where 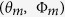 and 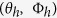 are the directional and azimuth angles for the magnetization (with saturation value of M s) and applied field vector (H), respectively, as shown in Fig. 4. The first term in eq. 1 represents the Zeeman energy in the applied magnetic bias field, and the effective anisotropy is the second term. The effective anisotropy energy, 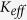 in its most general form, comes from the shape, magneto-crystalline and/or a magneto-elastic anisotropy^1^. Considering a single cylinder, the effective uniaxial anisotropy parallel to the wire axis is given by the shape demagnetization energy 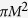 and a second-order uniaxial anisotropy contribution 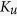. Additionally, the dipolar interaction between the nanowires should be taken into consideration. The corresponding dipolar energy is 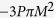, where P is the porosity (filling ratio) of the nanowire array, determined by the interwire distance R and wire diameter d:


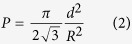


The average diameter of the FeNi nanowire is 80 nm, the average interwire distance is 100 nm and the porosity P is calculated to be 0.58. The additional second-order uniaxial anisotropy energy with the symmetry axis along the wire direction can be expressed as 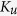. Taking these three factors into consideration, the effective anisotropy energy 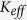 can be characterized by


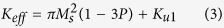


As a consequence, the corresponding intrinsic effective field 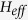, derived from the anisotropy energy, 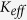, of the nanowire arrays can be expressed as


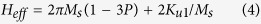


The ferromagnetic resonance frequency is obtained from the second derivative of the energy density in eq. 1 by the formalism of Smit and Beljers^23^ as


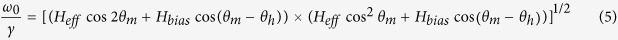


where 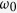 is the resonance frequency, 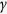 is the gyromagnetic ratio, defined positive 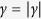, 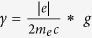, where 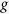 is the Landé factor, 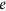 is the electronic charge, 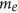 is the electronic rest mass, and c is the speed of light. For the geometry used in our experiment, the applied field varies in a plane of 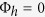 from 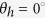 to 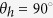 to the wire axis.

For NC at the saturated state, 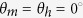, and the Kittel equation is


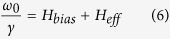


For PC at the saturated state, considering the bias field larger than the effective field, 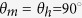,


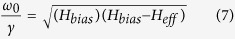


Fitting eqs 6 and 7 with different pumping frequencies (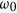) and the corresponding resonance fields (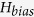) can give the gyromagnetic ratio (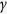) and the effective anisotropy field (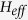) at NC and PC.

**Figure 5:**
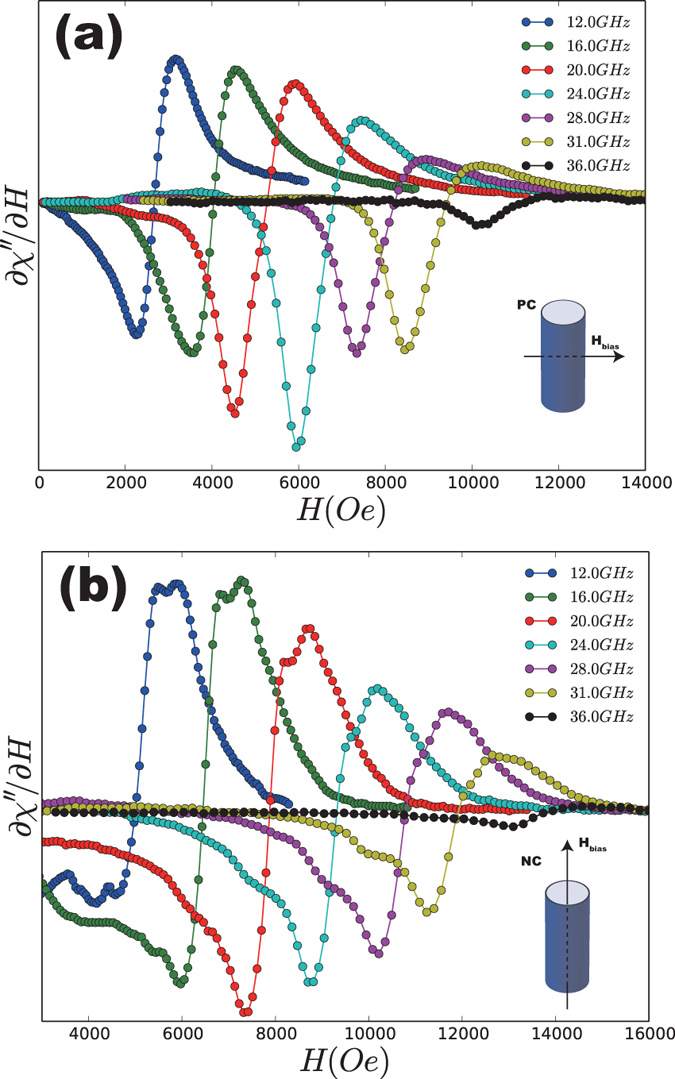
FMR derivative spectra for the FeNi gradient nanowire arrays at different pumping frequencies with the applied bias field (**a**) perpendicular to the nanowire axis (PC) and (**b**) parallel to the nanowire axis (NC).

Figure 5 shows the typical sequence of the gradient FeNi nanowire field-sweep FMR signals recorded from 12–38 GHz at PC (a) and NC (b). The experimental FMR absorption spectrum is proportional to the derivative of the absorbed power and 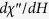 with respect to the applied field. The resonance fields and linewidths have been extracted by fitting the absorption curves with the Lorentz function. The spectra for PC (Fig. 5(a))are slightly different from those for NC (Fig. 5(b)). Substructures can be observed under NC and have also been observed in Ni nanowire arrays^16^. As shown in Fig. 5(b), the substructures differ with changing the pumping frequency, which could be influenced by the inhomogeneity of the pumping field. Additionally, because of the randomness of the structural disorder along the longitudinal of the wire axis, excited spin-waves with the wave vector parallel to the wire axis may lead to a broadening of the resonance lines^24^. The measured resonance absorption can be regarded as the integral of the absorption peaks of all individual wires. The variety of the structural disorder along the wire axis, generating the variation of wave vector 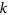, may also give rise to the substructure of the FMR absorption peaks.

**Figure 6:**
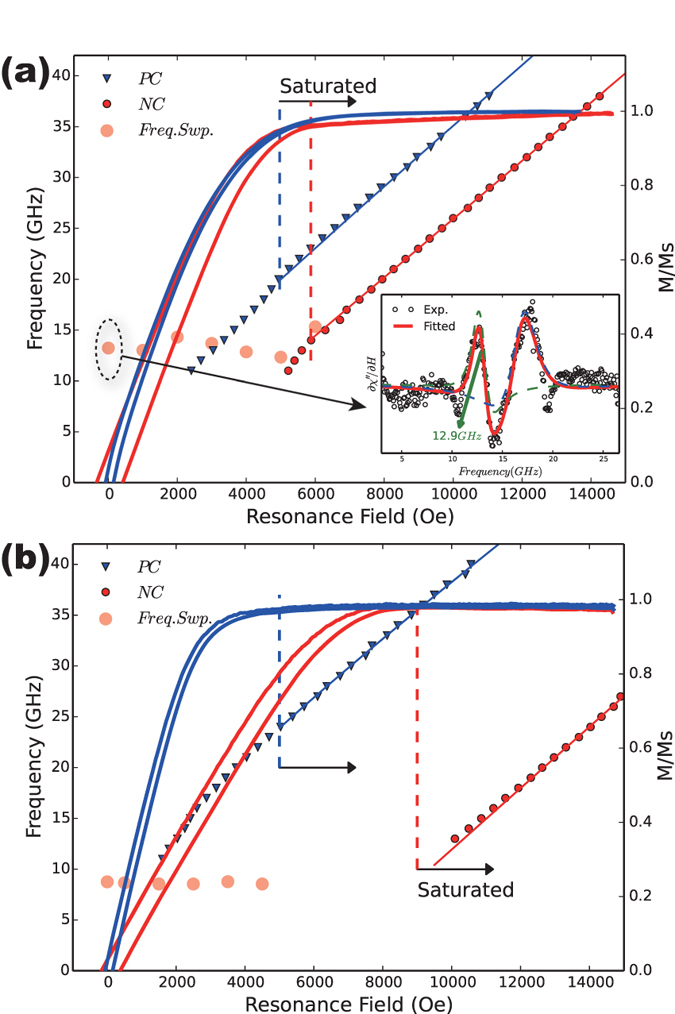
Field-sweep resonance field *vs*. the pumping frequency, along with the hysteresis loops under the PC and NC conditions for (**a**) the gradient FeNi nanowire and (**b**) the homogeneous FeNi nanowire. The lines show the expected behaviors from the Kittel resonance. The translucent circles indicate the resonance frequency *vs*. the applied bias field by frequency-sweep FMR. The inset of (**a**) is the zero applied field frequency-sweep spectra for the gradient nanowire array, fitted by two Lorenz functions.

The measured FMR resonance field 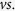 pumping frequency dispersion, along with the hysteresis loops for the gradient and homogeneous FeNi nanowires, are shown in Fig. 6(a,b), respectively. The dispersion relation indicates two different regions: the linear regions with 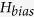 higher than the saturation field and the non-linear regions at a lower bias field. The dividing field of the two regions is consistent with the saturation field observed in the hysteresis loop. We used the data with a resonance field higher than the saturation field for fitting the Kittel equation (Eqs. 6 and 7). For NC at the saturated condition, the fitted Landé factor and intrinsic effective field for the gradient FeNi nanowire are 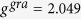 and 
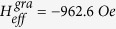
, respectively. For the homogeneous FeNi nanowire, 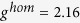 and 
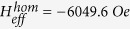
. Using Eq. 4, taking the magnetization saturation of 4
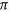
Ni_30_Fe_70_ M s = 10.2 kGauss^25^ into consideration, we can obtain the magneto-crystalline energy for the gradient FeNi nanowire, 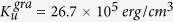, which is larger than that for the homogeneous FeNi nanowire, 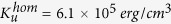. The results are smaller than the previous reports for the pure Ni nanowire values of 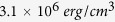 21. Because of the combination of the Fe atom and Ni atom, the magneto-crystalline energy for FeNi alloy will decrease. The enhancement of the magneto-crystalline anisotropy of the FeNi gradient nanowire may be due to the high textured structure, shown in Fig. 2, during deposition.

The fitted 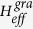 under PC (−4263.6 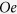) for the FeNi gradient nanowires is larger than that under NC. Two possible explanations are discussed below. First, an extra-high-order term, 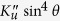, to the free energy may contribute to the magneto-crystal anisotropy or magnetoelastic anisotropy^26^ of the gradient nanowire. The contribution to the effective anisotropy field of this term is 0 when the applied bias is parallel to the nanowire axis. When the bias field becomes normal to the nanowire axis, 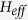 has an extra term of 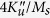 in the Kittel equation, shown in eq. 8.


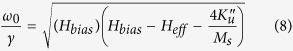


This phenomenon exists in the Co nanowires with the c-axis perpendicular to the nanowire axis^27^ and in Ni nanowires with high magneto-crystal anisotropy^28^. Considering the difference of 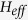 fitted under NC and PC, we can obtain the high magneto-crystal anisotropy of the FeNi gradient nanowire, 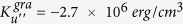. The high-order magneto-crystal anisotropy is a little larger than the reported Co nanowire of 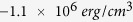. Another possible reason for the difference of the effective field under NC and PC may be the existence of a spin wave. The resonance we measured under PC may be a mixture of the uniform mode and the first spin wave mode. This means that the real uniform resonance field under PC may be higher than the resonance field that we measured. If we consider the effective field under PC and NC to be the same value, there will be an extra 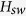 added to the resonance bias field. The extra field 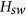 can be expressed as


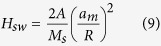


where A is the exchange stiffness. Considering 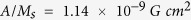 for NiFe alloy^29^ and an approximate analytic theory applicable for small pinning^30^, 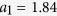 and 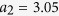. The nanowire radius (R) in our experiment is 40 nm. We can then obtain the extra field for the first spin wave, 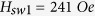, and the second spin wave extra field, 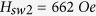. The mixture of the spin-wave resonance will reduce the PC resonance field, while the spin wave extra field is still smaller than the difference between the measured effective field from NC and PC FMR. Therefore, we assume that this phenomenon may be a mixed effect from the spin wave resonance and the high-order term of the uniaxial anisotropy.

It should be noted that the ferromagnetic resonance for nanowire arrays is more complex in the unsaturated region, which is related to not only the intrinsic effective field but also the remanent magnetization^31^. The field-sweep ferromagnetic resonance may change the magnetization state, which changes the shape of the spectra. Therefore, we used the frequency-sweep ferromagnetic resonance to investigate the unsaturated region to stabilize the magnetization inside the nanowire. The frequency-sweep measurements are performed for arrays by sweeping the frequency through the resonance peak for a fixed applied field parallel to the nanowire axis and strength. The inset in Fig. 6(a) represents the zero-field frequency-sweep ferromagnetic resonance spectra. The raw data are fitted with two Lorentz functions, as the nanowire arrays will exhibit two resonance peaks due to the remanent magnetization^31^,^32^. The first resonance frequency 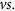 the applied bias field are scattered in Fig. 6. The points with the bias field near the saturation field are coincident with the field-sweep FMR results, indicating the same results for the field-sweep FMR and frequency-sweep FMR. By reducing the bias field, the resonance frequency remains at a constant value, as also noted by other researchers^1^,^16^.

**Figure 7:**
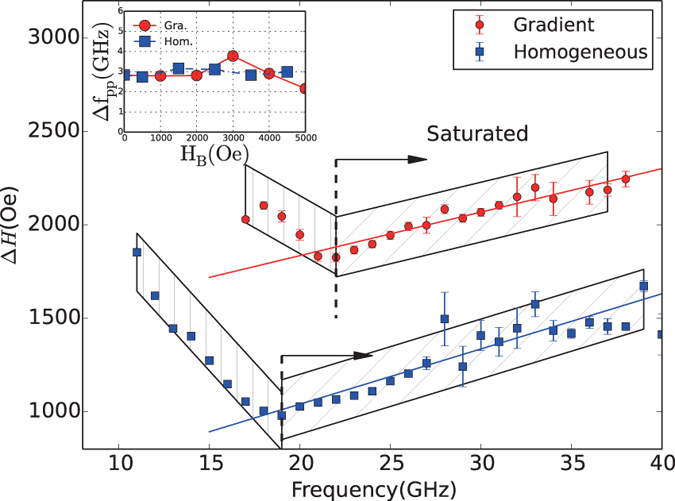
Field-sweep ferromagnetic resonance linewidth as a function of the pumping frequency. The right part indicates the single domain state. The inset is the frequency linewidth *vs*. the applied bias field by the frequency-sweep FMR.

Figure 7 shows the field sweep FMR linewidth 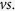 the pumping frequency with the applied bias field normal to the nanowire axis for the FeNi gradient (circle) and homogeneous (square) nanowires. The peak-to-peak spacing is measured as 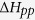. Two regions of the linewidths 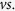 the pumping frequency are illustrated by the separating frequency at 22 GHz and 19 GHz for the gradient and homogeneous FeNi nanowires, respectively. The separating point corresponds to the saturated point shown in Fig. 6(a,b). The left part in Fig. 7 indicates that the nanowire array does not reach the single domain state, showing that the linewidth decreases with increasing pumping frequency. However, we also notice that the linewidth is not strictly proportional to the pumping frequency. The magnetization status, as well as the FMR spectra, changes significantly when sweeping the bias field lower than the saturation field^5^,^22^. To investigate the unsaturated linewidth, we plot the frequency-sweep FMR linewidth 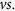 the fixed bias field lower than the saturation field to make the magnetization states inside the nanowire stationary. The inset of Fig. 7 is the frequency-sweep FMR linewidth 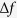 as a function of the applied bias field 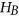. Interestingly, unlike the field-sweep FMR results, the frequency linewidths stay nearly constant at the multi-domain state. This phenomenon also exists in the uniform nanowires at the unsaturated state^33^. The motion of domain wall propagation^34^ may lead the linewidth to show an anomalous dispersion compared with films or bulks, which may need further investigation.

For the field-sweep FMR, when the pumping frequency increases, the linewidth shows a Gilbert type, 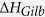, along with the inhomogeneous linewidth broadening, 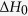.


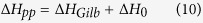



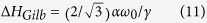


The Gilbert damping for the gradient FeNi nanowire is fitted to be 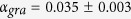, which is lower than the homogeneous FeNi nanowires, 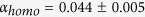. The reason for the decrease of the Gilbert damping for the gradient FeNi nanowire may be the decrease of the eddy current damping, 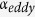, showing resistivity-like behavior^35^. The gradient alloy may have larger electrical resistivity compared with the homogeneous ones because of the pinning centers^10^, which reduce the eddy current in the gradient nanowires. It should be noted that the inhomogeneous broadening linewidth part, 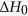, for the gradient FeNi nanowires is larger than that for the homogeneous ones. Because of the high concentration gradient, the crystal structure of the gradient FeNi nanowires differs along the longitudinal direction (as shown in Fig. 3(c,f)), leading an increase of the imperfection density. Surface pinning and two magnon-scattering effect^36^ may be heightened, which will enhance the inhomogeneous broadening.

## Discussion

In this paper, we examine the structure and magnetic behavior of the FeNi gradient composition binary nanowires grown in AAO templates.The XRD result shows that the nanowires are of face-centered cubic structures, with a highly textured structure of (110) orientation along the nanowire axis. According to the SAED pattern, the nanowire tends to form small grains at an Ni-rich area. With increasing Fe concentration, twinning fcc grains are observed. This may be caused by the high concentration gradient during deposition. The hysteresis loop along with the ferromagnetic resonance is used to obtain the intrinsic effective fields and damping. The gradient FeNi nanowires exhibit a stronger magneto-crystalline anisotropy than the homogeneous FeNi nanowires, which may be caused by the highly textured structure. According to the dispersion of the field-sweep linewidth 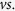 pumping frequency, the linewidth decreases with increasing pumping frequency. The frequency-sweep FMR linewidths stay nearly constant when the bias field is changed under the saturation field. When the applied bias field is enough to saturate the magnetic moments in the array, the linewidths exhibit the Gilbert type part, which is proportional to the pumping frequency. The Gilbert damping for the gradient FeNi nanowires is smaller than that for the homogeneous FeNi nanowires. The decrease of the eddy current damping, caused by the pinning centers, could be the reason for the decrease of the Gilbert damping. Because of the low Gilbert damping, the gradient FeNi nanowire array could be an alternative candidate for spintronics and microstrip antenna.

## Methods

The pulsed electrochemical method was used to fabricate the Fe/Ni gradient composition nanowires in commercial alumina templates with an average hole diameter of 80 nm. Before electrodepositing, one side of the AAO membrane was sputtered with a platinum layer as the working electrode. A platinum plate was used as the counter electrode. Electrodeposition was performed at room temperature in a three-electrode electrolytic cell in which the reference electrode was a saturated calomel electrode (SCE).

The electrolytic solution contained 1 mol/L NiSO_4_, 0.2 mol/L FeSO_4_ and 0.65 mol/L H_3_BO_3_. The pH value was maintained at 3.0. A 10 mA pulsed reduction current was used with a magnetic stirrer continuously agitated throughout the electrodeposition electrolyte to deposit the Ni and Fe compositions. In order to fabricate the gradient FeNi nanowires, first, the solution contained only Ni ionic liquid, and the FeSO_4_ was thn titrated into the solution at a certain rate. The homogeneous composite FeNi nanowires with the same diameter and length were also prepared using the standard electron deposition method for comparison.

Structural and morphological analysis of the nanowire arrays were performed using scanning electron microscopy (Apollo 300, JEOL JSM-7500F) and transmission electron microscopy (JEOL JEM-2100, LEOL JEM-2100F). The X-ray diffraction pattern of the sample was conducted on a Rigaku D/max2200PC using Cu K*α* radiation. The M-H loops were measured at room temperature with a vibrating sample magnetometer (VSM, BHV-50HTI) to study the hysteretic properties of the nanowires. The dynamic magnetic properties were characterized with variable-frequency ferromagnetic resonance using a coplanar wave guide.

## Additional Information

**How to cite this article**: Yang, H. *et al.* Static and Dynamic Magnetization of Gradient FeNi Alloy Nanowire. *Sci. Rep.* **6**, 20427; doi: 10.1038/srep20427 (2016).
